# A simple method for *ex vivo* honey bee cell culture capable of *in vitro* gene expression analysis

**DOI:** 10.1371/journal.pone.0257770

**Published:** 2021-09-23

**Authors:** Kazuyo Watanabe, Mikio Yoshiyama, Gaku Akiduki, Kakeru Yokoi, Hiroko Hoshida, Takumi Kayukawa, Kiyoshi Kimura, Masatsugu Hatakeyama

**Affiliations:** 1 Insect Gene Function Research Unit, Division of Insect Sciences, Institute of Agrobiological Sciences, NARO, Owashi, Tsukuba, Japan; 2 Animal Genetics Unit, Division of Animal Breeding and Reproduction Research, Institute of Livestock and Grassland Science, NARO, Ikenodai, Tsukuba, Japan; 3 Insect Pest Management Group, Division of Agro-Environment Research, Kyushu Okinawa Agricultural Research Center, NARO, Koshi, Kumamoto, Japan; 4 Insect Genome Research and Engineering Unit, Division of Applied Genetics, Institute of Agrobiological Sciences, NARO, Owashi, Tsukuba, Japan; USDA Agricultural Research Service, UNITED STATES

## Abstract

Cultured cells are a very powerful tool for investigating biological events *in vitro*; therefore, cell lines have been established not only in model insect species, but also in non-model species. However, there are few reports on the establishment of stable cell lines and development of systems to introduce genes into the cultured cells of the honey bee (*Apis mellifera*). We describe a simple *ex vivo* cell culture system for the honey bee. Hemocyte cells obtained from third and fourth instar larvae were cultured in commercial Grace’s insect medium or MGM-450 insect medium for more than two weeks maintaining a normal morphology without deterioration. After an expression plasmid vector bearing the *enhanced green fluorescent protein* (*egfp*) gene driven by the immediate early 2 (IE2) viral promoter was transfected into cells, EGFP fluorescence was detected in cells for more than one week from one day after transfection. Furthermore, double-stranded RNA corresponding to a part of the *egfp* gene was successfully introduced into cells and interfered with *egfp* gene expression. A convenient and reproducible method for an *ex vivo* cell culture that is fully practicable for gene expression assays was established for the honey bee.

## Introduction

Controlled *in vitro* cell systems, of both established cell lines and primary cultures, are valuable tools for understanding basic biological events by simplified host environment. Efforts to produce insect cell lines have resulted in the establishment of more than 1000 continuous cell lines from various insect species [1]. These cell lines are utilized for a broad range of biological research areas and the industrial mass production of recombinant proteins [2–4]. Nevertheless, cultured insect cells suitable for a specific research objective are not always available. In most cases, researchers use cell lines derived from closely related or commercially available species as an alternative or develop their own cell cultures [5].

The honey bee, *Apis mellifera* is one of the most important insects not only for honey production and its role as a pollinator of cultivated and natural plants, but also as a model insect for biological research, particularly on social behavior and learning. Decreases have recently been reported in managed honey bee colonies, and colony declines represent an ongoing concern that threatens the stable supply of pollinators worldwide [6–8]. One of the causes of colony declines is infections by viral pathogens [9–11]. *In vitro* systems, namely, primary cell cultures and cell lines, are valuable tools for investigating the mechanisms underlying interactions between viruses and host cells [2, 12]. Therefore, the development of honey bee cell cultures has been attempted, resulting in several primary cell cultures derived from a number of tissues and suitable culture media [13–15]. Only two stable cell lines have been established in the honey bee. One is the spontaneously immortalized cell line, AmE-711 [16] which has been utilized to investigate viral infection dynamics [17]. The other is the transformed cell line (MYN9) immortalized by the introduction of the human *c-myc* proto-oncogene [18]. However, these cell lines were infected with viruses [15, 17, 18].

Increased interest in the genetic regulatory mechanisms of the honey bee, *A*. *mellifera* has led to whole genome sequencing [19, 20] and the use of *in vivo* systems, such as gene knockdown by RNA interference (RNAi) [21], transposon-mediated germline transformation [22], and targeted mutagenesis (gene knockout) by genome editing [23–25]. Nevertheless, difficulties have been associated with experiments involving the individuals of eusocial species. Only a few studies have reported *in vitro* systems to induce gene expression through the introduction of expression vectors in honey bee cultured cells [18, 26]. To obtain a more detailed understanding of gene functions, the need for *in vitro* systems that enable examinations of gene interactions and their regulatory signal transduction pathways is increasing.

In the present study, we describe a simple, convenient, and reproducible method for an *ex vivo* honey bee hemocyte cell culture derived from the larval hemolymph. Commercial culture media, transfection reagents and expression vectors were compatible with cultivation, the introduction of exogenous DNA, and induction of gene expression in cultured cells. Moreover, the application of double-stranded RNA (dsRNA) to cultured cells successfully interfered with the expression of the target gene. This *ex vivo* cell culture system will provide new options for the study of honey bee molecular biology.

## Materials and methods

### Insects

Embryos, larvae, and pupae of the *Italian* honey bee, *Apis mellifera ligustica* were collected from colonies maintained in the research apiary of the Institute of Livestock and Grassland Science, NARO (Tsukuba, Japan).

### Culture medium and supplements

Commercially available Grace’s insect medium, Supplemented (Thermo Fisher Scientific, Waltham, MA, USA) containing 10% heat-treated fetal bovine serum (FBS, Corning, Corning, NY, USA) and MGM-450 insect medium [27] containing 20% FBS were used. Both culture media were supplemented with 10% antibiotics (Antibiotic-Antimycotic, Thermo Fisher Scientific).

### Preparation of honey bee cells and culture conditions

**Fig 1:**
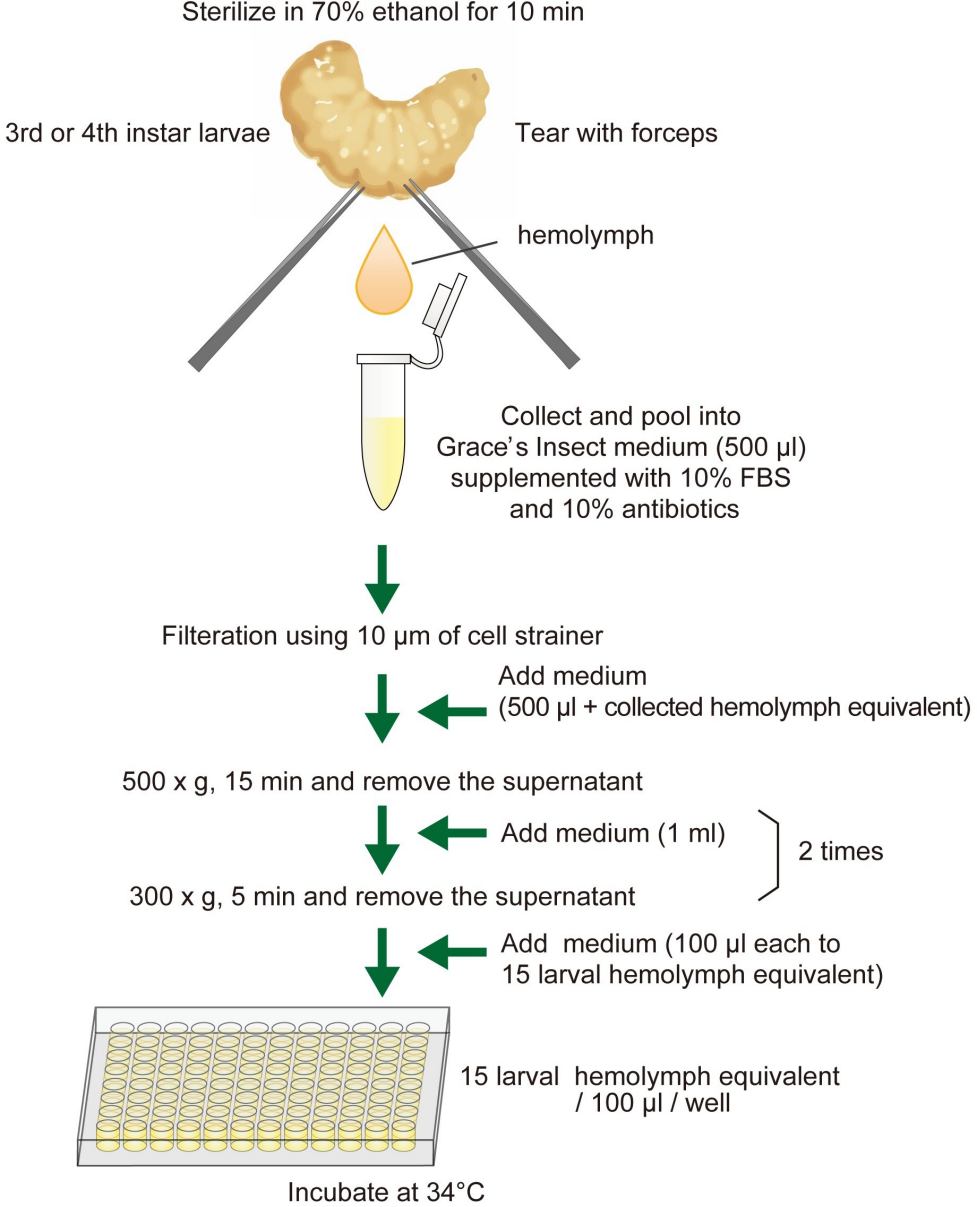
Method for culturing hemocyte cells.

The honey bee cells to be cultured were obtained from the hemolymph, embryos and fat bodies. To collect hemolymph cells, third or fourth instar larvae were carefully removed from comb cells using ring tip forceps, surface-sterilized with 70% ethanol for 10 min, and air-dried on a sterilized paper towel in a laminar flow hood. The dorsal abdomen was torn with a pair of forceps under a stereomicroscope, and one to two droplets of the hemolymph per individual were recovered in a 1.5-ml microcentrifuge tube filled with 500 μl of culture medium (Fig 1). The medium containing hemolymph cells was filtrated using a cell strainer (pluriStrainer Mini 10 μm, pluriSelect, Leipzig, Germany) to remove fat bodies and other debris. The filtrate was mixed with approximately the same volume of medium and centrifuged at 500 x g for 15 min to collect cells. Cells were then suspended in 1 ml of culture medium and centrifuged at 300 x g for 5 min. This centrifugation step was repeated once more. The cells of 15 larval hemolymph equivalents were suspended in 100 μl of culture medium.

Embryos (approximately 100 on average per preparation) of various stages (66–72 hours after oviposition) were removed from comb cells using thin forceps, surface-sterilized with 70% ethanol for 5 min, and air-dried on sterilized filter paper in a laminar flow hood. They were transferred to a drop of culture medium and chorions were removed with forceps. Dechorionated embryos were transferred into a 1.5-ml microcentrifuge tube filled with 50 μl of culture medium and gently homogenized with a plastic pestle. The homogenate was filtrated using a 40-μm cell strainer (pluriStrainer Mini 40 μm, pluriSelect).

Pupae were removed from comb cells using ring tip forceps. Fat bodies were dissected from the sterilized pupal abdomen, transferred to a 1.5-ml microcentrifuge tube filled with 1 ml of medium, and tissue fragments were rinsed in culture medium. Recovered cells in 100 μl of culture medium were dispensed into a well of a 96-well tissue culture plate (Sumitomo Bakelite, Tokyo, Japan). Each 96-well tissue culture plate was sealed with vinyl tape and incubated at 34˚C.

1. Surface-sterilize third or fourth instar larvae with 70% ethanol for 10 min and air-dry.
2. Tear the dorsal abdomen with a pair of forceps and recover one to two droplets of the hemolymph per individual (a total of 15 individuals) in a 1.5-ml tube filled with 500 μl of Grace’s insect medium containing 10% FBS and 10% antibiotics.
3. Filtrate the medium containing hemocytes using a 10-μm cell strainer and centrifuge three times with fresh culture medium.
4. Suspend hemocytes in 100 μl of culture medium and dispense into a well of a 96-well tissue culture plate, seal with vinyl tape, and incubate at 34˚C.

### Microscopy

Cell viability was examined using the trypan blue exclusion assay as previously described [28] with slight modifications. Cells were re-suspended in culture medium (100 μl) in a well, and a small portion (3 μl) was mixed with an equal volume of 0.4% trypan blue solution (Fujifilm Wako Pure Chemical, Osaka, Japan) in a 1.5-ml microcentrifuge tube. The mixture (6 μl) was then transferred to a hemocytometer (Watson, Tokyo, Japan) and the number of viable (unstained) and dead (stained) cells was counted under a microscope (IX70, Olympus, Tokyo, Japan). Three replications (three wells) were made for each incubation period, and the wells from which cells were taken were sacrificed after each count. Cell viability was calculated by the ratio of viable cells to total cells.

Enhanced green fluorescent protein (EGFP) fluorescence was observed under a fluorescent microscope equipped with a GFP filter (BZ-8100, Keyence, Osaka, Japan). The EGFP-positive and -negative cells observed within randomly selected areas of 200 μm x 200 μm squares under a fluorescent microscope were photographed and counted to estimate transfection efficiency (EGFP-positive cells among viable cells). The number of cells in three square areas per well was counted and three replications (for three wells) were made for each measurement.

### Polymerase Chain Reactions (PCR) and sequence analysis

To identify the origin of cultured cells, a fragment of the *A*. *mellifera* mitochondrial *cytochrome-c oxidase subunit I* (*COI*) gene (GenBank: NM_001177490) was amplified using KOD One DNA polymerase (Toyobo, Osaka, Japan) with the universal primer set [29] (Table 1) and genomic DNA as the template. Genomic DNA was extracted from cultured cells and adult workers using the DNeasy Blood & Tissue kit (Qiagen, Hilden, Germany) according to the supplier’s protocol. PCR conditions were as follows: 30 cycles at 94˚C for 10 sec, 55˚C for 5 sec, 68˚C for 5 sec, and final extension at 68˚C for 10 min. PCR products were examined by agarose gel electrophoresis and each PCR product was cloned into the pCR4Blunt-TOPO vector using the Zero Blunt TOPO PCR Cloning kit for Sequencing (Invitrogen, Thermo Fisher Scientific). The nucleotide sequences of cloned DNA fragments were determined by dye-labeled cycle sequencing using the BigDye Terminator Cycle Sequencing kit v3.1 (Applied Biosystems, Foster City, CA, USA) and analyzed with a DNA sequencer (ABI 3130x1, Applied Biosystems).

**Table 1:** List of primers.

Primers		sequence (5´ -> 3´)
**COI universal primers**		
	LCO 1490	GGTCAACAAATCATAAAGATATTGG
	HCO 2198	TAAACTTCAGGGTGACCAAAAAATCA
**Gene-specific primers**		
	EGFP L	ACGTAAACGGCCACAAGTTC
	EGFP R	CGGCCGCTTTACTTGTACAGCTC
	Am ef-1α L	GGTGTGAAACAATTGATTGTTGGTG
	Am ef-1α R	AAGACGGAGAGCCTTGTCTGTA
**T7-flanking primers for dsRNA synthesis**		
	EGFP dsRNA L	TAATACGACTCACTATAGGGCGTGACCACCCTGACCTAC
	EGFP dsRNA R	TAATACGACTCACTATAGGGGTTCTTCTGCTTGTCGGCCA
	Am kmo dsRNA F	TAATACGACTCACTATAGGGAGAAGTCGTGGATGCGGATTTAG
	Am kmo dsRNA R	TAATACGACTCACTATAGGGAGATCCTGAGGCGTTTTCAACTT

#### Reverse Transcription PCR (RT-PCR)

RT-PCR was performed to examine gene expression. Total RNA was extracted from cultured cells using the RNeasy Mini kit (Qiagen) according to the supplier’s protocol. Short fragments from transcripts of the *egfp* gene (656 bp) and the *elongation factor-1α* gene of *A*. *mellifera* (GenBank: XM_006569890) (315 bp) were amplified using the QIAquick RT-PCR kit using primer sets (Table 1) and total RNA (20 ng/ 20 μl reaction) as the template. PCR conditions were as follows: 50˚C for 30 min, 95˚C for 15 min, 35 cycles at 94˚C for 45 sec, 65˚C for 30 sec, and 72˚C for 1 min followed by final extension at 72˚C for 10 min. PCR products were examined by agarose gel electrophoresis.

### Plasmid construction and transfection

Three types of expression vector plasmids were used for transfection. An expression vector carrying the *egfp* gene under the control of the immediate early 2 (IE2) viral promoter (IE2::EGFP) was previously constructed [30]. The plasmid pIZT-EGFP has the pIZT/V5-His backbone (Thermo Fisher Scientific, Waltham, MA, USA) and the fragment coding the *egfp* ORF was inserted downstream of the IE2 promoter. The plasmid carrying the *egfp* gene driven by the *Bombyx mori actin 3* gene promoter (BmA3::EGFP) was previously constructed [30]. The plasmid carrying the *egfp* gene driven by the *B*. *mori actin 3* and *Drosophila melanogaster heat-shock protein 70* gene promoters in tandem (BmA3::EGFP/Dmhsp70::EGFP) was a transformation vector for the sawfly, *Athalia rosae* [31]. These vector plasmids were proven to induce *egfp* gene expression in the cultured cells of lepidopteran species (IE2::EGFP and BmA3::EGFP) [30] and *D*. *melanogaster* (BmA3::EGFP/Dmhsp70::EGFP) [32]. Vector plasmids were extracted and purified from *Escherichia coli* host cells (DH5α) using the QIAGEN Plasmid Mini kit (Qiagen, Hilden, Germany) or Mini Plus Plasmid Extraction kit (Viogene, New Taipei City, Taiwan).

**Fig 2:**
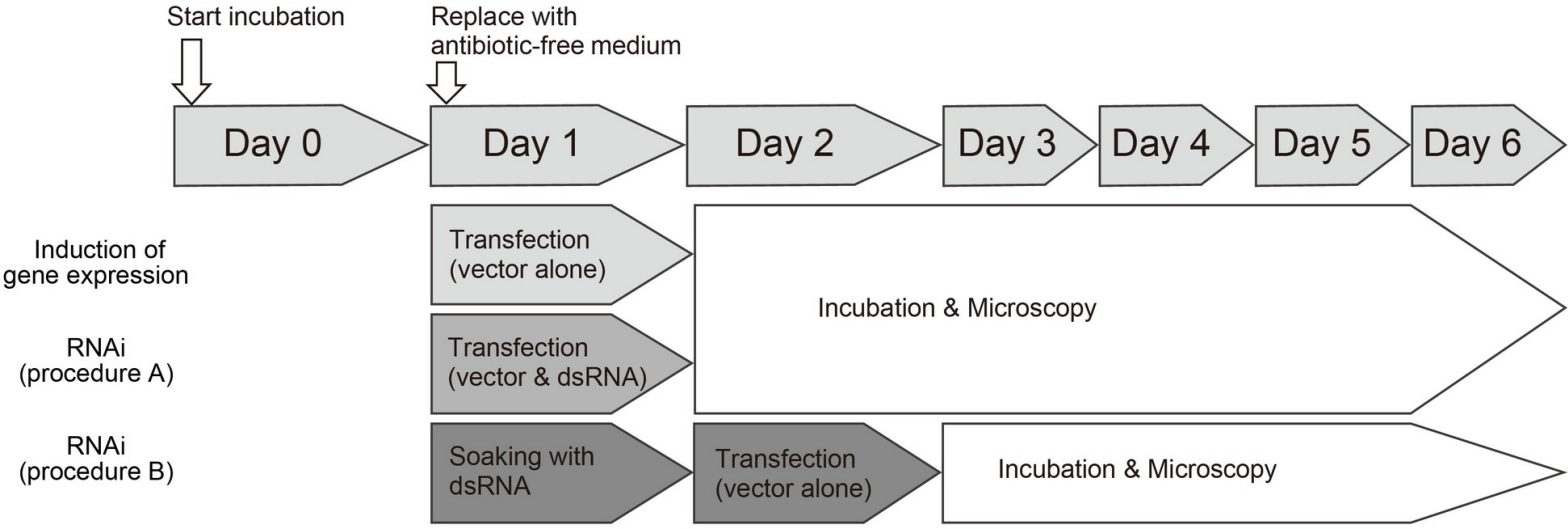
Manipulation timeline of the *ex vivo* cell culture. Days after incubation were referred to as Day 0, Day 1, Day 2, and thereafter. The cells on each day after the incubation were referred to as Day 1 cells, Day 2 cells, Day 3 cells, and thereafter. Experimental procedures for the induction or inhibition of gene expression are described below the time course. To induce gene expression, the expression vector plasmid was transfected into cells on Day 1. To inhibit gene expression (RNAi, procedure A), a mixture of the expression vector plasmid and dsRNA was transfected into cells on Day 1. In another procedure to inhibit gene expression (RNAi, procedure B), cells were incubated with dsRNA-containing culture medium on Day 1, incubated for one day, and the expression vector plasmid was then transfected into cells on Day 2.

One day after the incubation, Day 1 cells were subjected to transfection after culture medium had been replaced by antibiotic-free medium (Fig 2). Two transfection reagents, TransIT-Insect (Mirus, Bio, Corp., Madison, WI, USA) and FuGene HD (Promega, Madison, WI, USA) were used. The plasmid was mixed with 9 μl/well of serum-free culture medium and transfection reagent (transfection mixture). After an incubation at 25˚C for 30 min, the transfection mixture was applied to Day 1 cells in the wells and the plate was incubated at 34˚C.

### Gene knockdown by RNA interference (RNAi)

Short cDNA fragments of the *egfp* gene (298 bp) and *kynurenine 3-monooxygenase* (*kmo*) gene of *A*. *mellifera* (GenBank: XM_624243) (357 bp) were PCR-amplified using a PrimeStar HS DNA polymerase (Takara Bio, Shiga, Japan), gene-specific primers to which the T7 RNA polymerase promoter sequence was incorporated at the 5´ end (Table 1). *kmo* dsRNA was used as the control because the *kmo* mutation does not affect viability in several insects including hymenopteran species [33–35]. The template DNAs used were as follows: the BmA3::EGFP/Dmhsp70::EGFP plasmid carrying *egfp* ORF, and cDNA synthesized from the total RNA of workers in the early pupal stage using Prime Script II (Takara Bio). Each dsRNA was synthesized using an Ambion MEGAscript T7 kit (Ambion, Thermo Fisher Scientific) and the PCR-amplified T7-flanking DNA fragment as a template. The concentration of synthesized dsRNA was adjusted to 5 μg/μl and maintained at –20˚C until used.

dsRNA (1 μg/well) was added to the transfection mixture, incubated at 25˚C for 30 min, and applied to Day 1 cells (RNAi, procedure A, Fig 2). In another experiment (RNAi, procedure B, Fig 2), dsRNA alone was applied to Day 1 cells and incubated for one day, namely cells were soaked in dsRNA-containing culture medium for one day. Day 2 cells soaked in dsRNA-containing culture medium were then subjected to the transfection of the expression vector plasmid with transfection reagent as described above. Cells treated with dsRNA were examined for EGFP fluorescence under a fluorescent microscope three days after transfection (Fig 2), namely Day 4 cells for procedure A and Day 5 cells for procedure B, and total RNA was then extracted for a RT-PCR analysis.

## Results

### Viability of *ex vivo* cultured honey bee cells

**Fig 3:**
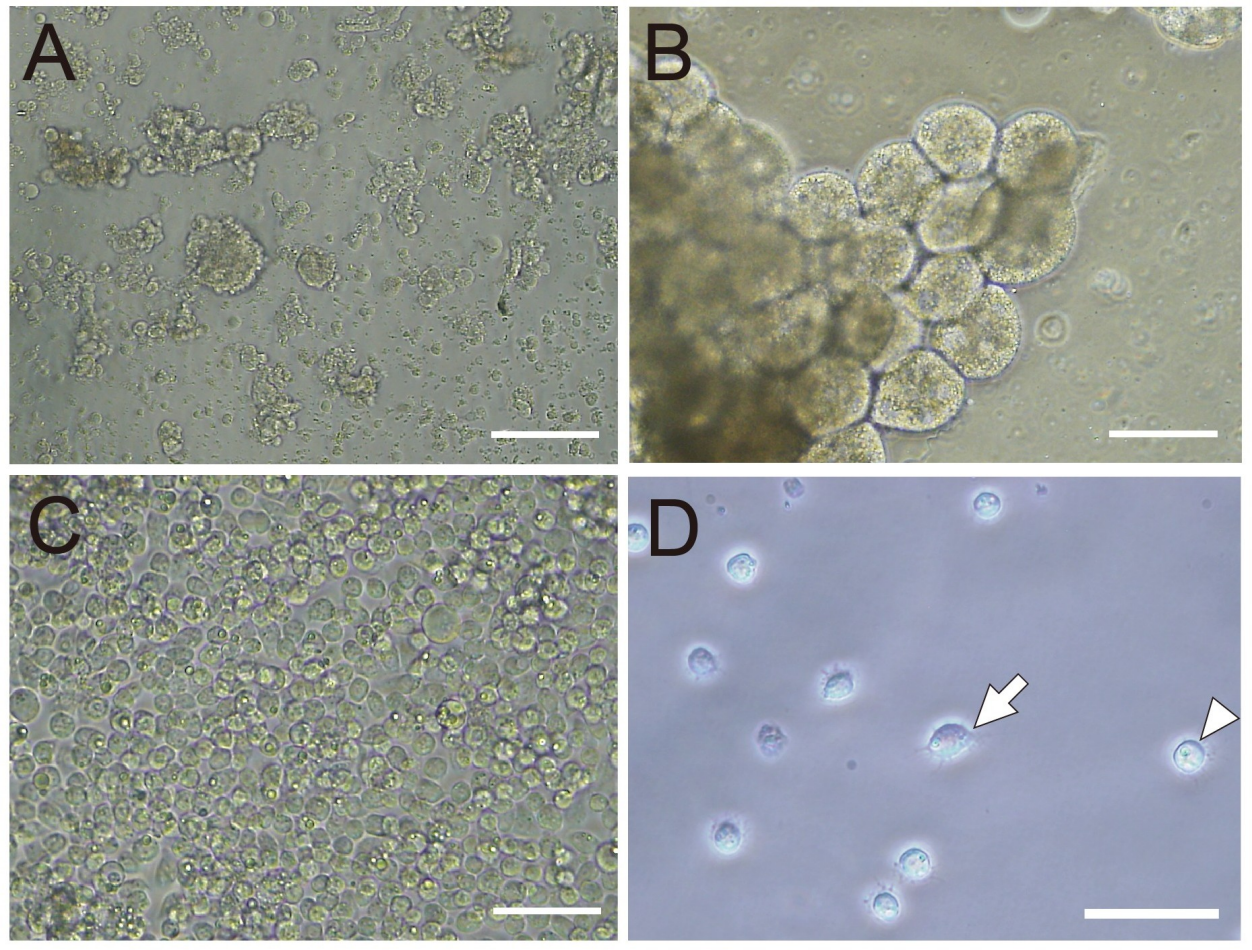
Morphology of honey bee cells prepared from each tissue. **A.** Embryonic cells derived from whole embryos. **B**. Pupal fat body cells were aggregated and irregular in shape and size. **C**. Hemolymph-derived cells were adherent and relatively uniform in shape and size. **D**. A magnified image of larval hemocytes. The arrow and arrowhead indicate a plasmatocyte and granulocyte, respectively. Bars indicate 100 μm.

**Fig 4:**
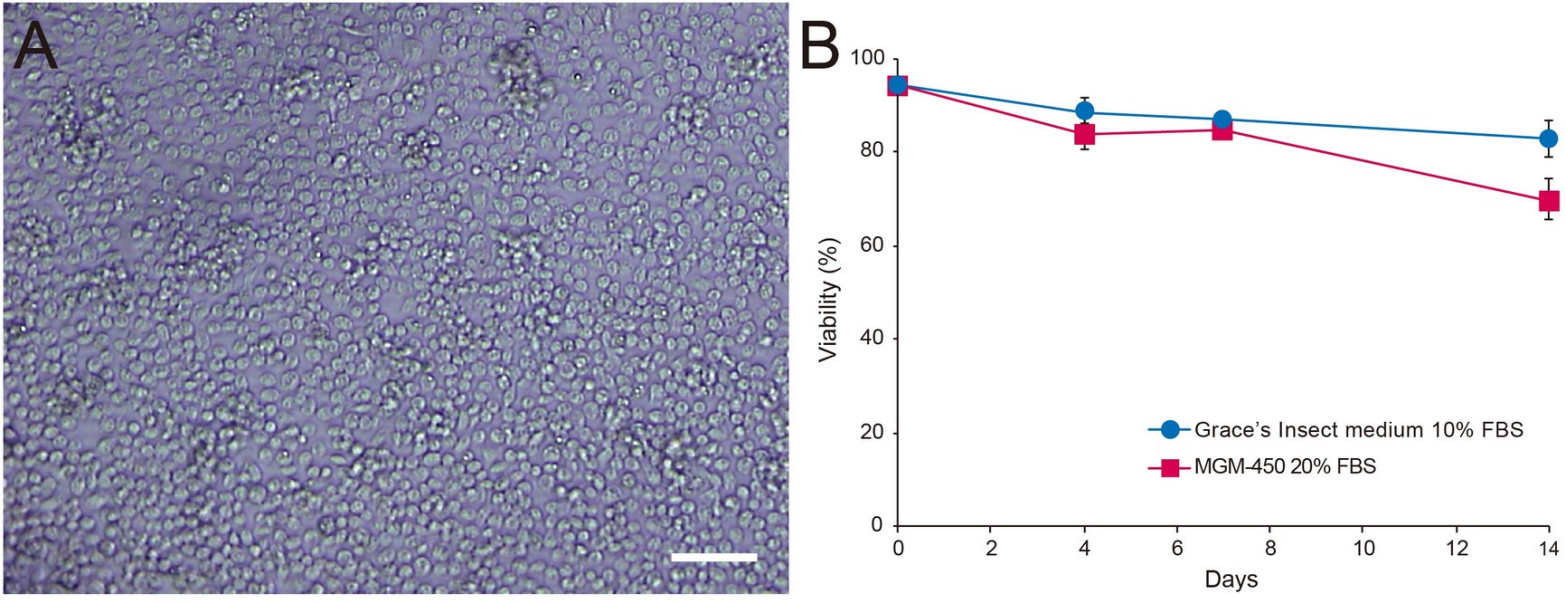
*Ex vivo* cultured hemocyte cells and viability after incubation. **A**. Hemocyte cells obtained from the hemolymph of 15 larval equivalents were fully confluent (approximately 4.45 x 10^5^ cells/well) in a well of a 96-well plate. The bar indicates 100 μm. **B**. Viability of cells after the incubation. Viability is the ratio of viable cells among all cells counted using a hemocytometer (three replications for each incubation period). Error bars indicate the standard error of the mean.

**Fig 5:**
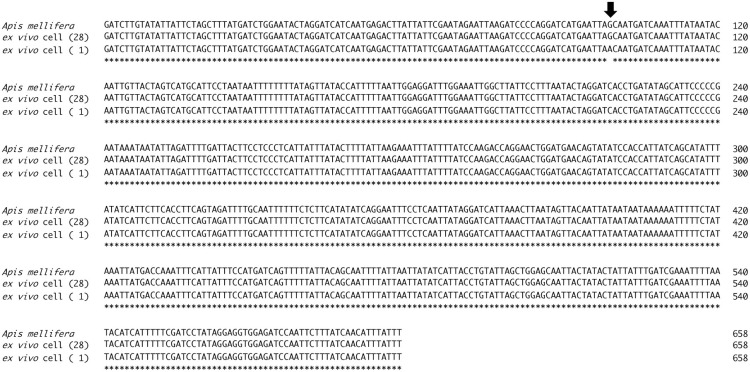
Alignment of nucleotide sequences of the mitochondrial *cytochrome c oxidase subunit I* (*COI*) gene. A partial sequence of the *Apis mellifera COI* gene (658 bp excluding the primer sites) is shown on the top. Twenty-eight out of the 29 clones obtained from *ex vivo* cultured cells had the same sequences to *A*. *mellifera COI* (middle line). One clone (bottom line) had a single base substitution (arrow). Asterisks indicate identical bases.

Among the cells prepared from the larval hemolymph, embryos and fat bodies, hemolymph-derived cells were suitable for further cultivation because they were uniform in size and adherent (Fig 3). In contrast, embryonic cells and fat bodies were both difficult to manipulate due to their overall characteristics. Embryonic cells were a mixture of cells derived from various tissues and inefficient to recover because the total number of viable cells to introduce into the culture was low. Fat body cells were aggregated and irregular in shape and size, in addition to the difficulty of recovery from pupae. Hemolymph-derived cells had the characteristics of adherent hemocytes consisting of plasmatocytes and granulocytes. Filopodia, which are required to adhere to foreign surfaces, were observed. Spreading-plasmatocytes had spindles (Fig 3D, arrow) and granulocytes were round in shape (Fig 3D, arrowhead) [36–38]. Hemocytes fully confluent in a well of a 96-well plate (15 larval hemolymph equivalents/well, or approximately 4.45 x 10^5^ cells/well) were cultured at 34˚C in Grace’s insect medium containing 10% FBS or MGM-450 medium containing 20% FBS supplemented with 10% antibiotics. More than 80% of cells cultured in Grace’s insect medium and approximately 70% of those in MGM-450 medium were viable after 14 days (Fig 4). Hemocyte cells were successfully cultured in 33 out of 36 replicates, and cells in the three unsuccessful cases were contaminated and eventually died. We investigated whether these cells originated from the honey bee was examined by the PCR amplification of a mitochondrial *cytochrome c oxidase subunit I* (*COI*) gene fragment (709 bp). The sequences of 29 clones from four independent cultures were determined, and 28 completely matched that of *A*. *mellifera*, while one had a single-base substitution. These results confirmed the cells were of *A*. *mellifera* origin (Fig 5).

### Induction of gene expression

**Fig 6:**
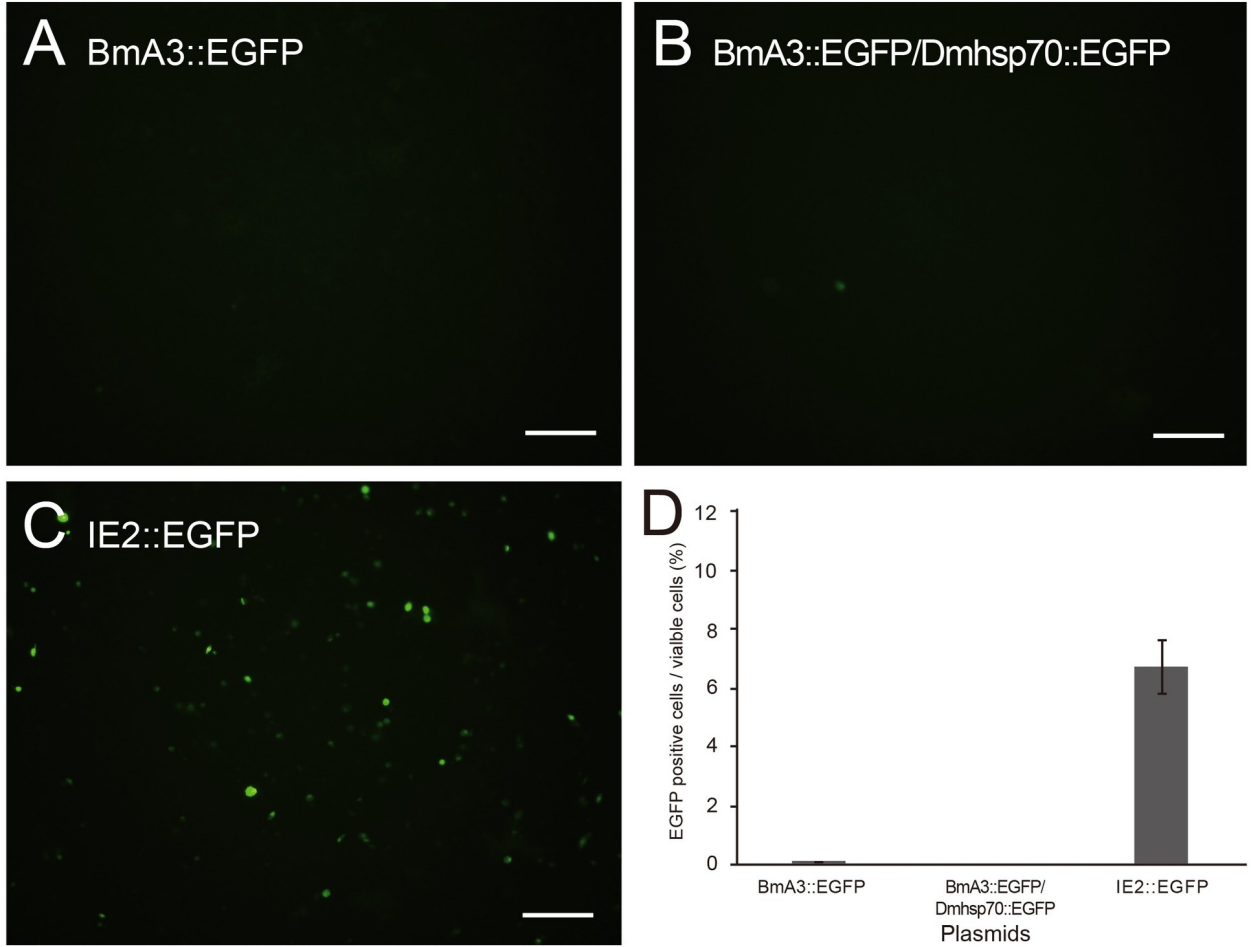
Induction of *egfp* gene expression by the introduction of expression vector plasmids into *ex vivo* cultured cells. Fluorescent micrographs of Day 4 cells transfected with expression vectors of **A**. BmA3::EGFP, **B**. BmA3::EGFP/Dmhsp70::EGFP, and **C**. IE2::EGFP. The bar indicates 100 μm. **D**. EGFP fluorescence was predominantly detected in cells transfected with the IE2::EGFP vector plasmid. Error bars indicate the standard error of the mean in three replications.

**Fig 7:**
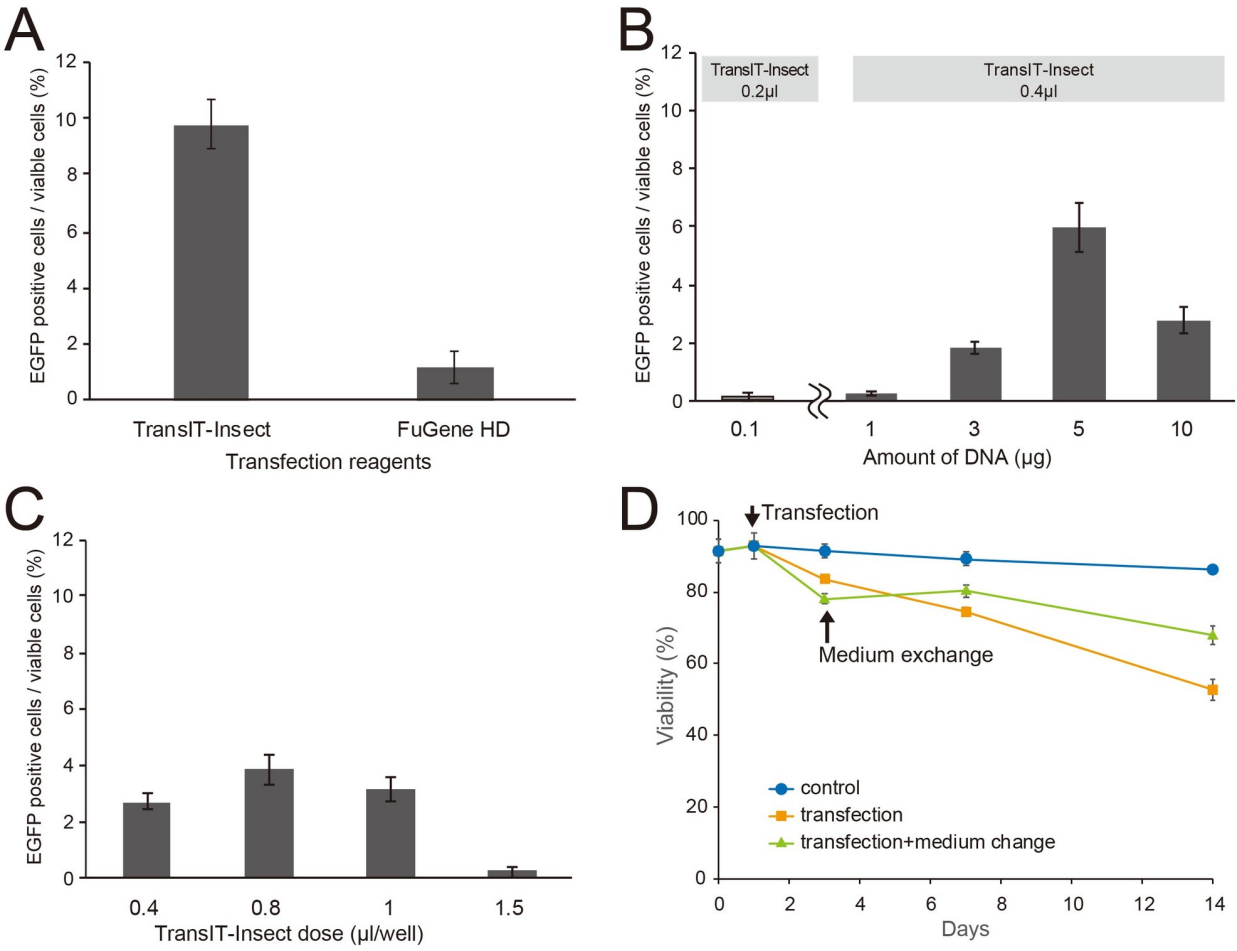
Selection of optimal conditions for the induction of gene expression. **A**. Comparison of transfection reagents. **B**. The optimal amount of vector plasmid DNA to be transfected was 5.0 μg/well when 0.4 μl/well of the TransIT-Insect reagent was applied. The graph on the left shows transfection efficiency when the conditions recommended by the supplier were employed. **C**. The optimal dose of the transfection reagent was 0.8 μl/well when 5.0 μg/well of plasmid DNA was applied. **D**. Effects of medium exchange after transfection on cell viability. Error bars indicate the standard error of the mean of three replications.

Three types of expression vector plasmids were subjected to transfection. All plasmids carried the *egfp* gene as a reporter under the control of different promoters: the *immediate early 2* (IE2) viral promoter (IE2::EGFP), the *Bombyx mori actin3* gene promoter (BmA3::EGFP), and the *B*. *mori actin3 and Drosophila melanogaster heat-shock protein 70* gene promoters in tandem (BmA3::EGFP/Dmhsp70::EGFP). Day 1 cells were transfected with each plasmid using transfection reagents. EGFP fluorescence was detected one day after transfection through Day 14, only when the IE2-driven *egfp* gene vector (IE2::EGFP) was used (Fig 6). It is noted that there was no background autofluorescence in control (untreated) cells (S1 Fig). The number of cells emitting EGFP fluorescence peaked on Day 4–5 or 3–4 days after transfection. Transfection efficiency in terms of the proportion of cells exhibiting EGFP in Day 5 cells was higher when cells were cultured in Grace’s insect medium than in MGM-450 insect medium. TransIT-Insect showed higher transfection efficiency than FuGene HD (Fig 7A).

We then selected the optimal conditions for inducing gene expression using cells cultured in Grace’s insect medium and transfected with the IE2::EGFP vector plasmid using the TransIT-Insect reagent. The number of EGFP-positive cells was small when the recommended protocol of the TransIT-Insect reagent (0.1 μg of plasmid DNA with 0.2 μl of the TransIT-Insect reagent) was applied to Day 1 cells for transfection (Fig 7B, left). Day 1 cells were then transfected with different amounts of plasmid DNA (1.0, 3.0, 5.0 and 10.0 μg) using 0.4 μl of the TransIT-Insect reagent (Fig 7B, right). EGFP-positive cells became detectable when the amount of Trans-IT-Insect reagent was doubled (0.4 μl) and plasmid DNA was more than 1.0 μg. The proportion of EGFP-positive cells increased when 5.0 μg of plasmid DNA was applied, while the viability of cells was not affected by the amount of the plasmid added. Plasmid DNA (5.0 μg) was then applied to Day 1 cells using different volumes (0.4, 0.8, 1.0 and 1.5 μl) of the TransIT-Insect reagent. The proportion of EGFP-positive cells was the highest when 0.8 μl of TransIT-Insect was used; however, the addition of more than 1.0 μl of the TransIT-Insect reagent reduced the proportion of EGFP-positive cells (Fig 7C). The exchange of medium on the day after the addition of the TransIT-Insect reagent promoted cell survival because an excess amount of the TransIT-Insect reagent is toxic to cells (Fig 7D). Therefore, to induce gene expression, cells incubated in Grace’s insect medium need to be transfected with 5.0 μg of the IE2::EGFP vector plasmid using 0.8 μl of the TransIT-Insect reagent. Under optimal conditions, transfection efficiency (EGFP-positive cells among viable cells) was 7.78% ± 0.49 (mean ± SE) (16 replications).

### Inhibition of gene expression by RNAi

**Fig 8:**
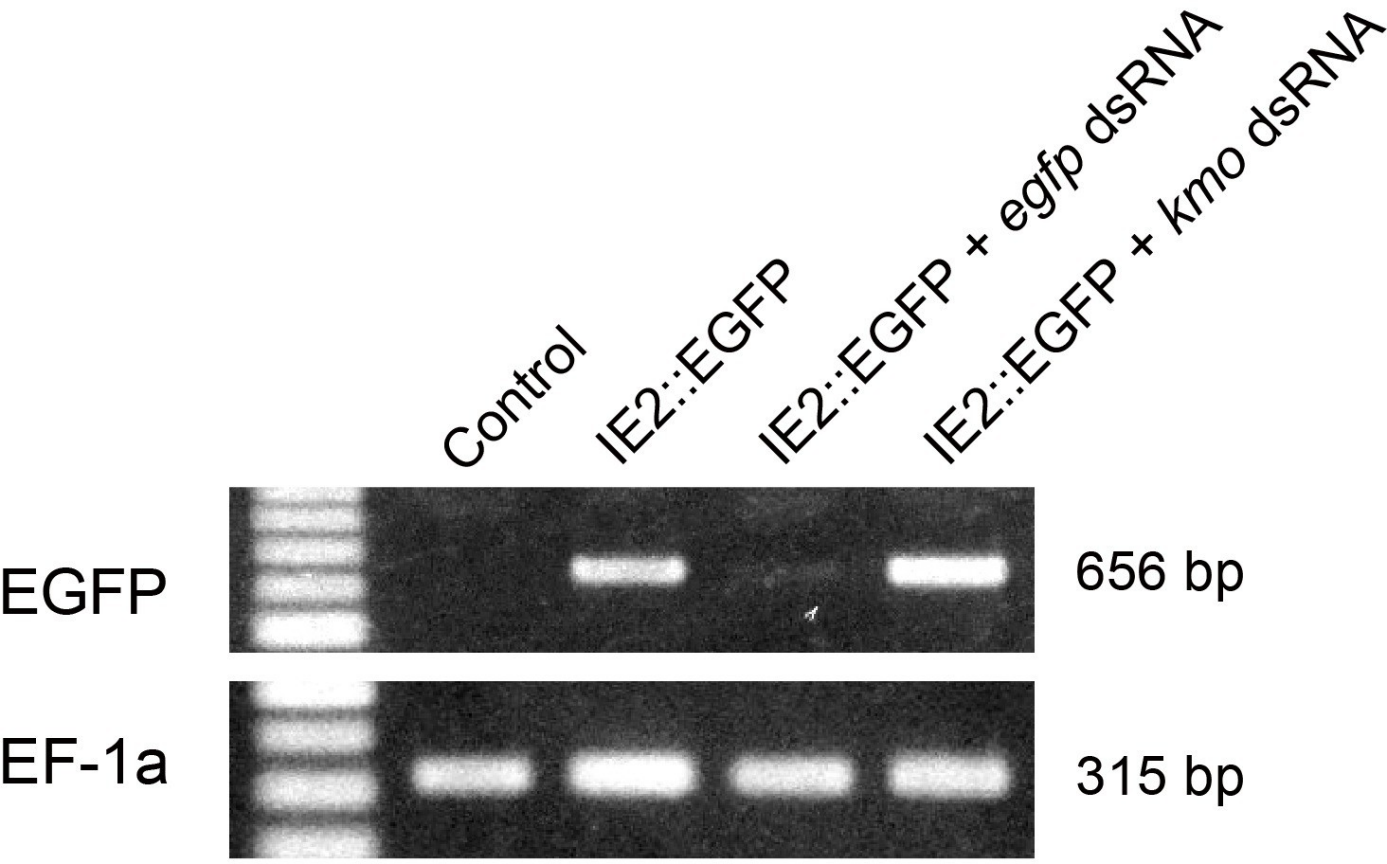
Comparison of transcripts levels by RT-PCR. Short fragments of the *egfp* gene (656 bp, top panel) and *A*. *mellifera elongation factor-1α* gene (315 bp, bottom panel) were amplified using gene-specific primer sets and total RNA extracted from Day 5 cells as the templates. *egfp* gene transcripts were detected in cells transfected with the IE2::EGFP vector, but not in cells to which dsRNA corresponding to the *egfp* gene was applied. Each lane indicates the treatments that *ex vivo* cells received: no treatment (Control), transfection of the IE2::EGFP vector (IE2::EGFP), transfection of the IE2::EGFP vector associated with *egfp* dsRNA (IE2::EGFP + *egfp* dsRNA), and transfection of the IE2::EGFP vector associating with dsRNA corresponding to an unrelated *A*. *mellifera kynurenine 3-monooxygenase* (*kmo*) gene (IE2::EGFP + *kmo* dsRNA).

**Fig 9:**
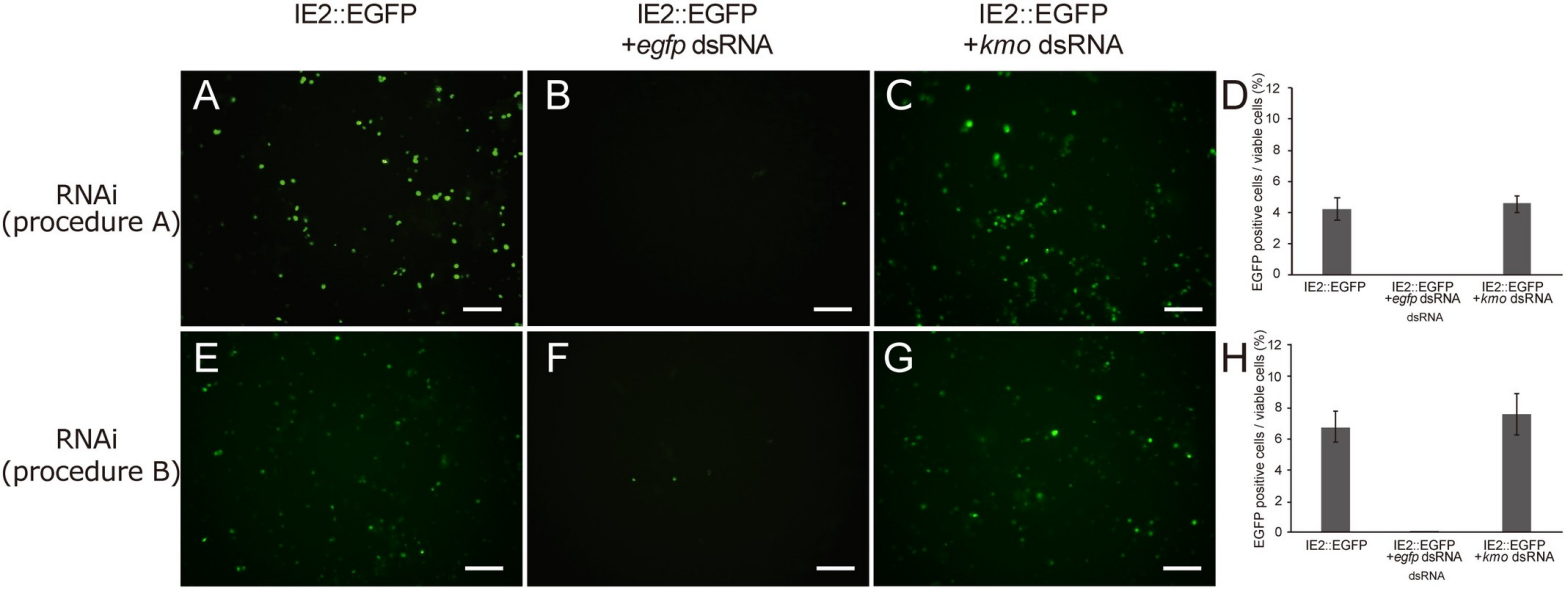
Fluorescent micrographs showing a decrease in EGFP emissions by RNAi-based gene silencing in *ex vivo* cultured cells. Top panels (**A**-**C**) show the effects of the application of dsRNA using the transfection reagent (RNAi, procedure A), and bottom panels (**E**-**G**) show the effects of soaking with dsRNA without transfection reagent (RNAi, procedure B). *Ex vivo* cells were transfected with the IE2::EGFP vector (**A** and **E**, IE2::EGFP), transfected with the IE2::EGFP vector and the application of *egfp* dsRNA (**B** and **F**, +*egfp* dsRNA), and transfected with the IE2::EGFP vector and application of *kmo* dsRNA (**C** and **G**, +*kmo* dsRNA). **D**, **H**. The transfection of and soaking with *egfp* dsRNA both decreased the proportion of EGFP-positive cells. Bars indicate the standard error of the mean of three replications.

dsRNA corresponding to a part of the *egfp* gene was synthesized and applied to the cell culture. The IE2::EGFP vector plasmid and *egfp* dsRNA or control *kmo* dsRNA were mixed and introduced into Day 1 cells using the TransIT-insect reagent (RNAi, procedure A). The expression of the *egfp* gene was examined on Day 4, namely, three days after application. The *egfp* transcript was maintained at a lower level than in cells devoid of *egfp* dsRNA (Fig 8). The proportion of EGFP-positive cells also markedly declined (Fig 9). *egfp* transcripts and the proportion of EGFP-positive cells were unaffected by the application of *kmo* dsRNA. We then examined whether *egfp* dsRNA was taken up by cultured cells without the transfection reagent when it was added to culture medium (RNAi, procedure B). *egfp* dsRNA was added to Day 1 cells and soaked for a day, and the IE2::EGFP vector plasmid was transfected into cells using the TransIT-Insect reagent on the next day (Day 2). The level of the *egfp* gene transcript remained low and EGFP fluorescence was only detected in a small proportion of cells, similar to when the mixture of the plasmid vector and *egfp* dsRNA was transfected into cells (Fig 9).

These results indicated that gene expression in *ex vivo* cultured cells was inhibited by RNAi-mediated gene silencing regardless of the presence of a transfection reagent. The gene knockdown effect persisted for at least one week after the introduction of dsRNA.

## Discussion

**Fig 10:**
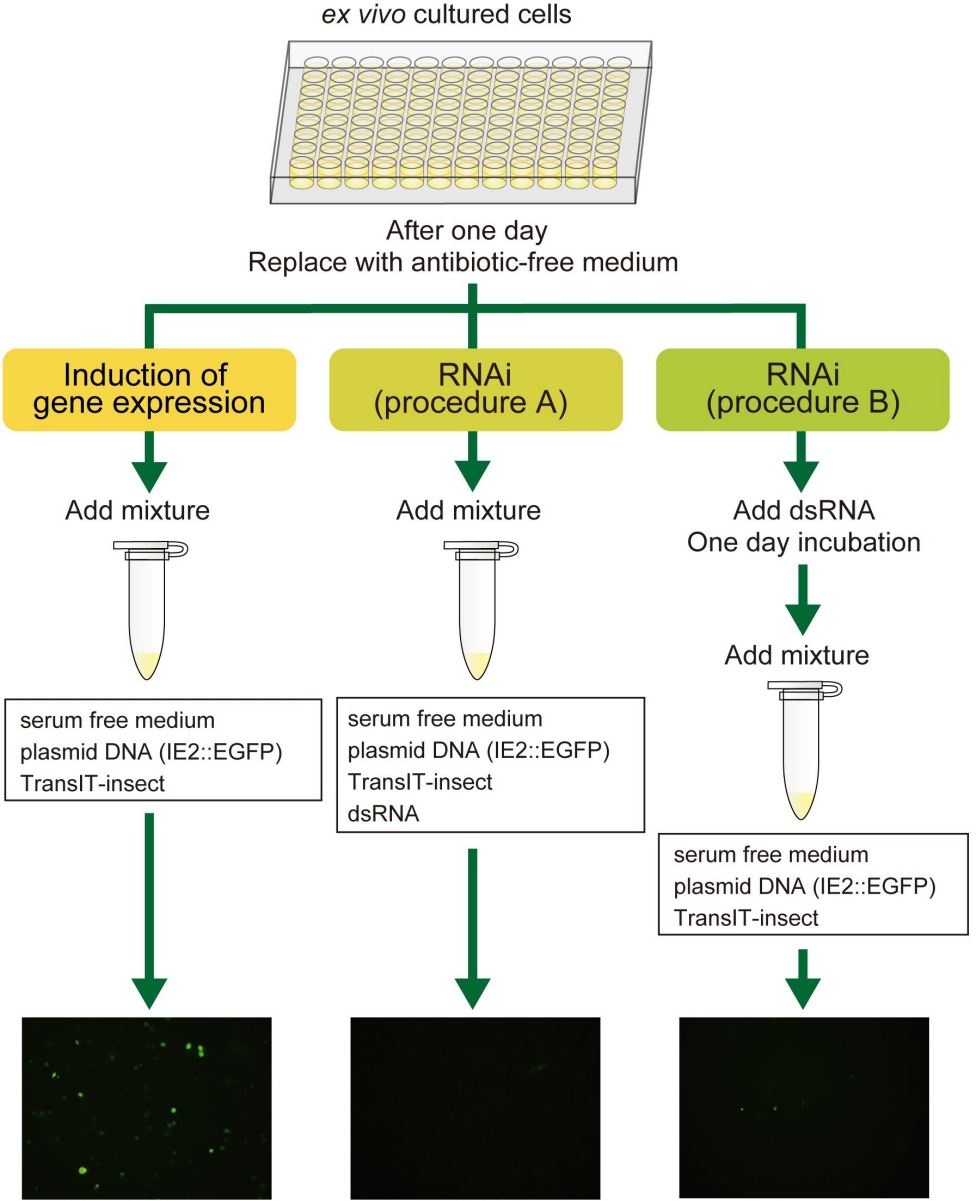
Procedure for inducing and inhibiting gene expression in *ex vivo* cultured hemocyte cells under optimal conditions.

*Ex vivo* cultured hemocyte cells are capable of inducing gene mis-expression (induction and suppression) and serve as an *in vitro* system for a gene functional analysis of the honey bee (Figs 1 and 10). Commercially available culture media and transfection reagents are available for these experiments. Although continuous cell lines are desirable for *in vitro* experiments, particularly virus-cell interactions in the honey bee, only two cell lines, both derived from embryos, have been reported to date: one is an established continuous cell line (AmE-711) [16] and the other is an engineered cell line (MYN9) by the introduction of a human nuclear proto-oncogene (*c-myc*) [18]. Difficulties are associated with maintaining these cell lines, which may be due to viral infection [15, 17, 18]. In contrast, primary cultured cells have been established from various tissues and organs in the honey bee, and these primary cell cultures are proven to be useful for viral studies and neuronal processes [15, 39, 40]. Although primary cell cultures and *ex vivo* cultured cells may only be maintained for a limited period of time under healthy conditions, these cells are available for examining gene expression and signaling pathways [2, 5]. The present results demonstrating the induction of gene mis-expression indicate that *ex vivo* cultured hemocyte cells will be a practical option for an *in vitro* system for the honey bee. Gene expression was evidently induced, and transcripts were detectable after PCR amplification; however, the proportion of the cells in which EGFP fluorescence was detected was low. Mosquito cell lines with low transfection efficiency have been used for luciferase reporter gene assays [41]. Reporter gene assays are highly sensitive, and generally feasible as long as the expression vectors may be transfected into a portion of the cultured cells. *Ex vivo* cultured hemocyte cells, even with low transfection efficiency, may be used for these assays. Possible applications of *ex vivo* cultured hemocyte cells will involve the study of factors (*cis* and *trans* elements) that influence transcriptional activity and their utilization as material for RNA-seq after induction of conditional expression. *Ex vivo* cultured hemocyte cells may be used to examine susceptibility to chemical reagents such as insecticides, in combination with reporter gene assays. They will also be applicable to the screening of effective genome editing tools, CRISPR/Cas9, through examinations of DNA-digesting efficiency by inducing the expression of the vector bearing guide RNA and cDNA of nuclease.

One day after the incubation (Day 1), replace the culture medium with antibiotic-free medium.

Induction of gene expression (left):

1. Mix 5.0 μg of the IE2::EGFP vector plasmid and 0.8 μl of the TransIT-Insect reagent in 9 μl of serum-free culture medium (transfection mixture) and incubate at 25˚C for 30 min.
2. Apply the transfection mixture to Day 1 cells in a well and incubate at 34˚C.
3. Replace culture medium with fresh medium on the next day (Day 2) and incubate at 34˚C.

Inhibition of gene expression by the application of dsRNA at the same time as transfection (middle, RNAi, procedure A):

1. Mix 5.0 μg of the IE2::EGFP vector plasmid, 0.8 μl of the TransIT-Insect reagent and 1 μg of *egfp* dsRNA in 9 μl of serum-free culture medium and incubate at 25˚C for 30 min.
2. Apply the mixture to Day 1 cells in a well and incubate at 34˚C.
3. Replace culture medium with fresh medium on the next day (Day 2) and incubate at 34˚C.

Inhibition of gene expression by the soaking of dsRNA prior to transfection (right, RNAi, procedure B):

1. Apply 1 μg of *egfp* dsRNA to Day 1 cells in a well and incubate at 34˚C for one day.
2. Mix 5.0 μg of the IE2::EGFP vector plasmid and 0.8 μl of the TransIT-Insect reagent in 9 μl of serum-free culture medium (transfection mixture) and incubate at 25˚C for 30 min.
3. Apply the transfection mixture to Day 2 cells in a well and incubate at 34˚C.
4. Replace culture medium with fresh medium on the next day (Day 3) and incubate at 34˚C.

The successful RNAi-based gene silencing of *ex vivo* cultured cells was achieved by simply soaking with dsRNA in culture medium. Gene silencing by dsRNA-mediated RNAi in cell cultures has been exclusively performed using established continuous cell lines [42]. Following the success of soaking RNAi in *Drosophila* S2 cells [43], this method has been applied to other insect cell lines, such as lepidopteran Sf21 originating from *Spodoptera frugiperda* and SL-1 from *S*. *litura*, Tc81 and TcA from the coleopteran *Tribolium castaneum*, and Lepd-SL1 from the coleopteran *Leptinotarsa decemlineata* [44–47]. The functional uptake of dsRNA achieving RNAi-based gene silencing occurred by the soaking method in these cell lines. Similarly, dsRNA in culture medium was taken up by *ex vivo* cultured honey bee hemocyte cells. It is important to note that insect hemocytes are more likely to be sensitive to dsRNA uptake than other tissues, even in species in which systemic RNAi is not recognized [48–51]. Previous studies demonstrated that phagocytic hemocytes in *D*. *melanogaster* are specialized in the uptake of dsRNA by endocytosis, which is mediated by scavenger-like pattern-recognition receptors [52, 53]. RNAi-based gene silencing *in vivo* by the injection of dsRNAs into the adult body cavity is less effective in the honey bee because tissue-dependent dsRNA uptake seems to be limited to fat body cells [54]. However, cultured cells are free from the constraints imposed in *in vivo* environments that interfere with dsRNA uptake, such as nucleases degrading dsRNA in the hemolymph [55] and macromolecules competing with scavenger-like receptors for the binding of dsRNA [52]; therefore, *ex vivo* hemocyte cells are susceptible to RNAi, which also reflects its original physiological characteristics. The transfection of dsRNA into a cell culture is an alternative to the soaking method and effectively induces intracellular RNAi in *ex vivo* cultured honey bee hemocyte cells, similar to the continuous cell lines of Sf21 and CiE1 from the lepidopteran *Chrysodeixis includens* [56, 57].

Commercially available culture media and transfection reagents are compatible with the *ex vivo* honey bee cell culture method described here. The viral promoter, IE2, which is often used in versatile expression vectors, induced constitutive gene expression; however, the promoters of different insect species (*Bombyx actin3* and *Drosophila heat-shock protein 70*) did not work in the present study. Some promoters of honey bee genes have been shown to drive conditional gene expression in the Sf21 cell line [58], and are assumed to function in *ex vivo* cultured honey bee cells. The manipulation of endogenous gene expression will enable analyses of the functions of promoters, cis regulatory elements, transcriptional factors, and signal transduction.

We selected hemocyte cells for the *ex vivo* culture primarily because of their adherence and ease of handling. Insect hemocytes play crucial roles in the cellular immune system [59]. Specific cellular reactions, such as phagocytosis, micro-aggregation, nodulation, and encapsulation, have been examined in detail [59, 60]; however, there are relatively few studies on the honey bee [61, 62]. The molecular mechanisms underlying insect cellular immunity remains unclear [2]. Since the *in vitro* system of insect hemocytes that retains their original characteristics is a requisite for examining the essential processes of cellular immune reactions, attempts to develop hemocyte primary cell cultures have been made in several insects, including the honey bee [40, 63–68]. Primary cultured honey bee hemocytes could be infected with sacbrood virus and Flock House virus, which replicate intercellularly and peak in abundance 72–96 hours after infection [40]. Our method to enable the control of gene expression in *ex vivo* cultured honey bee hemocyte cells is simple and reproducible. A functional analysis of candidate genes involved in the cellular immune response will be possible using *ex vivo* cultured honey bee hemocytes through the combination of viral infection and RNAi-based gene silencing. The method described here may be applicable to other insect species and provides an *in vitro* system not only for immunological examinations, but also a wide range of insect studies.

## Supporting information

S1 Fig Autofluorescence was undetectable in *ex vivo* cultured hemocyte cells. Top panels show phase-contrast micrographs of **A.** untreated (intact) *ex vivo* cultured hemocyte cells and **C.** cells transfected with expression vectors of IE2::EGFP. Bottom panels show the micrographs of **B**. untreated cells and **D.** cells transfected with expression vectors of IE2::EGFP. Background autofluorescence was detected neither in untreated (intact) cells nor *egfp* expression-induced cells. All cells represent Day 7 cells. Bars indicate 100 μm.
(PDF)

S2 Fig Raw agarose gel images for Fig 8. Gel images were taken using a FUNA-BOX Imaging System (FBOX-03Sir, Funakoshi, Tokyo, Japan) equipped with a digital camera (XZ-2, Olympus, Tokyo, Japan) and UV transilluminator (M10E, UVP, Upland, CA USA). Images were cropped and labeled using Adobe Photoshop Elements 15 software.
(PDF)
