# Supplementary figures and images for: A simple method for ex vivo honey bee cell culture capable of in vitro gene expression analysis
Source: PLoS One. 2021 Sep 23;16(9):e0257770. doi: 10.1371/journal.pone.0257770 (PMC8460014; doi:10.1371/journal.pone.0257770)

S1 Fig. Autofluorescence are undetectable in *ex vivo* cultured hemocyte cells.

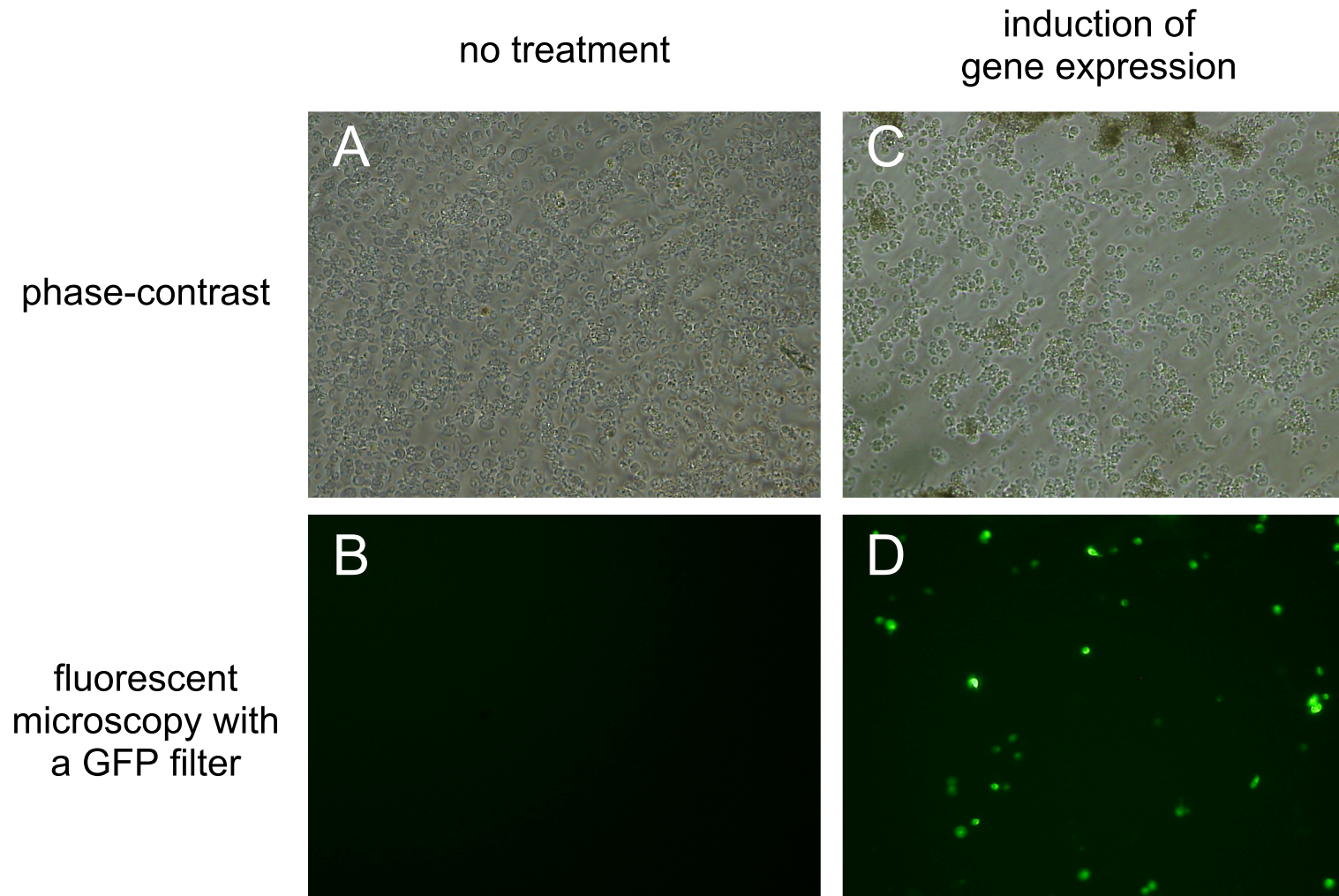

Supplement: S1 Fig — Top panels show phase-contrast micrographs of A. untreated (intact) ex vivo cultured hemocyte cells and C. cells transfected with expression vectors of IE2::EGFP. Bottom panels show the micrographs of B. untreated cells and D. cells transfected with expression vectors of IE2::EGFP. Background autofluorescence was detected neither in untreated (intact) cells nor egfp expression-induced cells. All cells represent Day 7 cells. Bars indicate 100 μm. (PDF) [file pone.0257770.s001.pdf]
