# Supplementary material for: A simple method for ex vivo honey bee cell culture capable of in vitro gene expression analysis
Source: PLoS One. 2021 Sep 23;16(9):e0257770. doi: 10.1371/journal.pone.0257770 (PMC8460014; doi:10.1371/journal.pone.0257770)
Supplement: S2 Fig — Gel images were taken using a FUNA-BOX Imaging System (FBOX-03Sir, Funakoshi, Tokyo, Japan) equipped with a digital camera (XZ-2, Olympus, Tokyo, Japan) and UV transilluminator (M10E, UVP, Upland, CA USA). Images were cropped and labeled using Adobe Photoshop Elements 15 software. (PDF) [file pone.0257770.s002.pdf]

Raw agarose gel images used for Fig. 8

EGFP

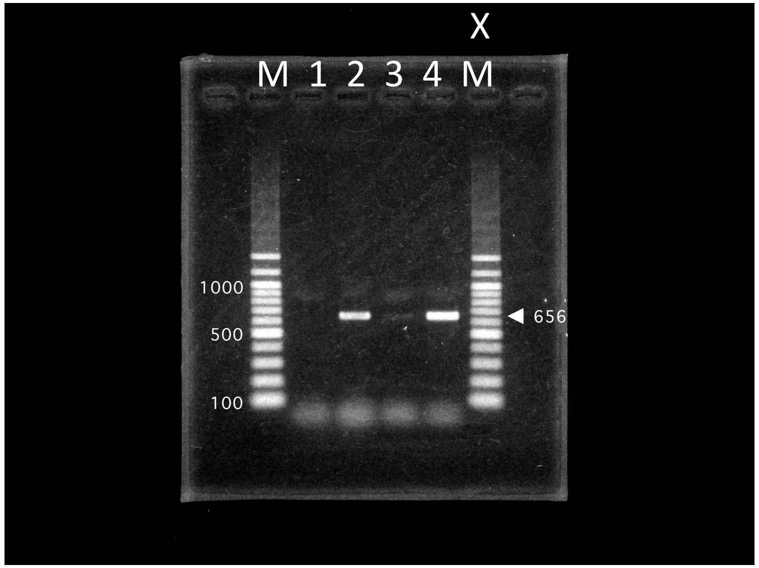

Amplicons of *egfp* fragment

M: size marker (100 bp ladder)
1: Control (no treatment)
2: IE2::EGFP transfected
3: IE2::EGFP transfected with *egfp* dsRNA
4: IE2::EGFP transfected with *kmo* dsRNA

EF-1α

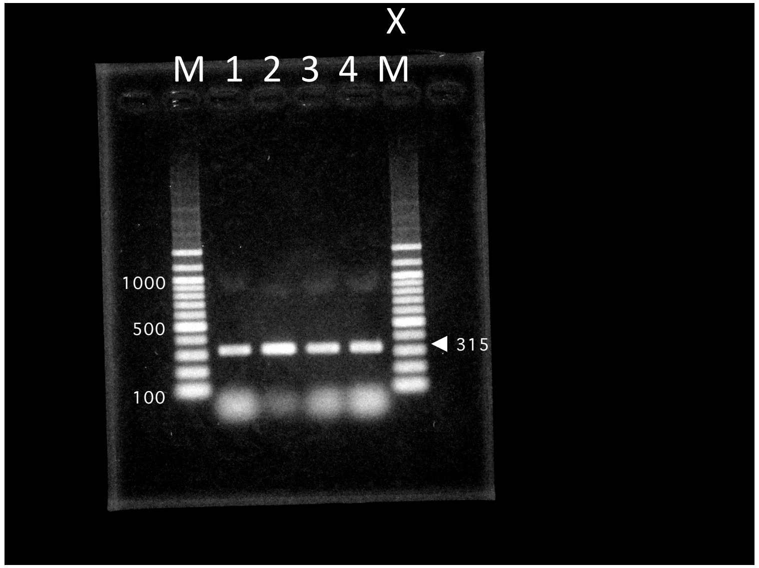

Amplicons of *A. mellirera ef-1α* fragment

M: size marker (100 bp ladder)
1: Control (no treatment)
2: IE2::EGFP transfected
3: IE2::EGFP transfected with *egfp* dsRNA
4: IE2::EGFP transfected with *kmo* dsRNA

Gel images were taken using a FUNA-BOX Imaging System (Funakoshi, FBOX-03Sir) equipped with a digital camera (Olympus, XZ-2) and a UV transilluminator (UVP, M10E). The images were cropped and labelled using an Adobe Photoshop Elements 15 software.
